# An efficient and robust hybrid method for segmentation of zebrafish objects from bright-field microscope images

**DOI:** 10.1007/s00138-018-0934-y

**Published:** 2018-05-10

**Authors:** Yuanhao Guo, Zhan Xiong, Fons J. Verbeek

**Affiliations:** Imaging & BioInformatics, LIACS, Leiden University, Niels Bohrweg 1, 2333 CA Leiden, The Netherlands

**Keywords:** Zebrafish segmentation, Bright-field microscope, Hybrid method, Mean shift algorithm, Level set method, High-throughput imaging

## Abstract

Accurate segmentation of zebrafish from bright-field microscope images is crucial to many applications in the life sciences. Early zebrafish stages are used, and in these stages the zebrafish is partially transparent. This transparency leads to edge ambiguity as is typically seen in the larval stages. Therefore, segmentation of zebrafish objects from images is a challenging task in computational bio-imaging. Popular computational methods fail to segment the relevant edges, which subsequently results in inaccurate measurements and evaluations. Here we present a hybrid method to accomplish accurate and efficient segmentation of zebrafish specimens from bright-field microscope images. We employ the mean shift algorithm to augment the colour representation in the images. This improves the discrimination of the specimen to the background and provides a segmentation candidate retaining the overall shape of the zebrafish. A distance-regularised level set function is initialised from this segmentation candidate and fed to an improved level set method, such that we can obtain another segmentation candidate which preserves the explicit contour of the object. The two candidates are fused using heuristics, and the hybrid result is refined to represent the contour of the zebrafish specimen. We have applied the proposed method on two typical datasets. From experiments, we conclude that the proposed hybrid method improves both efficiency and accuracy of the segmentation of the zebrafish specimen. The results are going to be used for high-throughput applications with zebrafish.

## Introduction

**Fig. 1:**
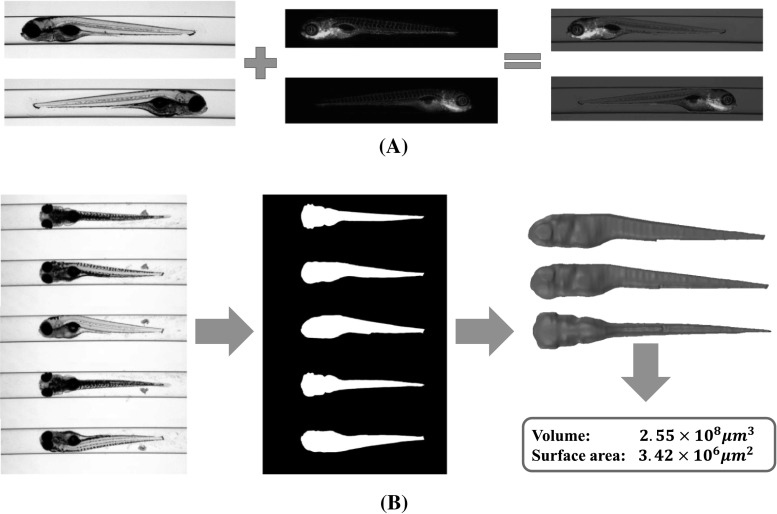
Typical applications of zebrafish segmentation. **a** Fluorescence images visualisation and evaluation. Bright-field zebrafish images offer reference for the shape of the specimen (column one). Fluorescent images present informative signals, e.g. the blood vessels in green (column two). Accurate segmentation of the bright-field image provides a good shape reference to evaluate the fluorescent signals, for example, the development and concentration of specific cells (column three). **b** 3D zebrafish reconstruction from axial views. Axial-view zebrafish images (column one) are segmented to obtain 2D binary shapes (column two), from which the axial-view-based 3D reconstruction produces 3D models as well as 3D measurements (column three) (colour figure online)

High-throughput imaging applications pose a challenge to the image acquisition in that in some cases the quality of the imaging is compromised at the cost of the speed of the imaging. Often, this compromise is well studied and the loss of quality is relatively mild. We have studied high-throughput applications for zebrafish; the zebrafish is a popular model system in biomedical research. At present, high-throughput applications for zebrafish can be found, among others, in the fields of toxicology, cytology and oncology [1, 2].

The development of zebrafish high-throughput imaging [3] has resulted in massive amounts of data, i.e. images, becoming available. This requires an *efficient* and *robust* analysis for the images, so that phenotype descriptions of the zebrafish can be generated. Genetically engineered zebrafish can be labelled with fluorescent markers. Images from fluorescence present good properties of visibility and measurability for cancer cells and organs. In order to evaluate the features which are usually represented as colour intensity and concentration from the fluorescence, accurate segmentation of the zebrafish in bright-field images is quite essential to offer a shape reference for the measurements [4]. So, feature evaluations from control and experimental groups become comparable. In Fig. 1a, an example of this application is depicted.

Moreover, we can observe more informative features, e.g. volume, surface area and 3D shape variation, in 3D zebrafish imaging [5]. To this end, we need accurate 2D zebrafish segmentation to obtain sufficient shape priors for the axial-view-based 3D zebrafish reconstruction [6]. In Fig. 1b, we show this application.

In a particular case, according to the observation that the hemopoietic stem cells in zebrafish predominantly distribute in the tail, an accurate description of the overall shape of the zebrafish will ensure the evaluation of particular diseases by detecting and localising the tail region [7, 8]. Thus, an accurate segmentation of zebrafish objects in bright-field microscopy is very significant for a large range of biomedical applications.

**Fig. 2:**
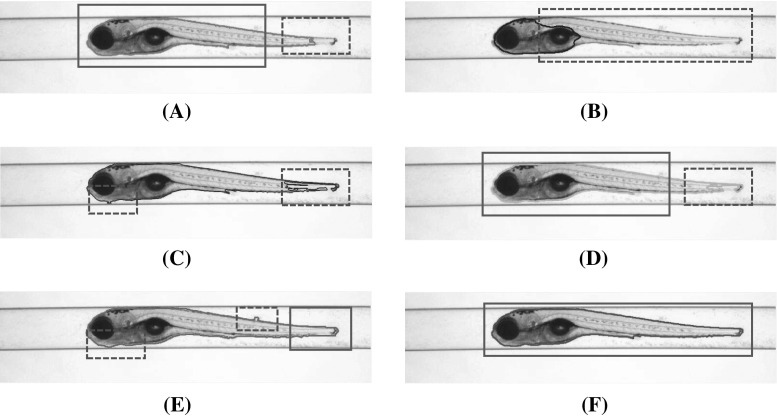
Segmentations by different methods for a zebrafish specimen in lateral position. Blue bounding box indicates the expected segmentations, and red bounding box indicates inaccurate segmentations. **a** Segmentation by the geodesic active contours (GAC) model. Due to the edge sensitivity, the GAC model fails to detect the tail of the specimen. **b** Segmentation by Chan–Vese (CV) model. The partial transparency of the specimen makes it difficult for a region-based method to discriminate the object from the background. **c** Segmentation by a local region-based level set (LRLS) model. Similar problem occurs that the tail of the specimen is incorrectly segmented. **d** Segmentation by an improved level set (ILS) method. **e** Segmentation by mean shift (MS) algorithm. Better results are obtained though; edge sensitivity becomes worse. **f** Segmentation by the proposed hybrid (HY) method. The accurate segmentation presents a natural and compact shape description for the zebrafish specimen (colour figure online)

Computational methods from the field of computer vision can, in principle, help to accomplish the image segmentation task in zebrafish imaging. However, when popular image segmentation methods are applied, for example, the geodesic active contours (GAC) model [9] and the Chan–Vese (CV) model [10], the inhomogeneity of the intensity distribution caused by partial transparency and edge discontinuity of zebrafish larvae usually results in an inaccurate segmentation. To illustrate these effects, in Fig. 2a, b, the segmentation results from, respectively, the GAC model and the CV model are shown. These segmentations show that the CV model converges at the most observable region, but fails to retain the whole shape of the object; the GAC model obtains a poor shape description for the zebrafish tail. As shown in Fig. 2c, d, other improved algorithms, such as the local region-based level set (IRLS) model [11] and the improved level set (ILS) method [12], also do not result in an accurate segmentation of the zebrafish.

In fact, the edge-based methods including the GAC model and the ILS method are able to accurately discriminate the visible edges, but suffer from the problem of edge leakage. In contrast, as depicted in Fig. 2e, unsupervised learning methods such as the mean shift (MS) algorithm [13] can obtain an overview shape description for the object, while the explicit edge will be, to a certain extent, contaminated due to region fusion effects.

**Fig. 3:**
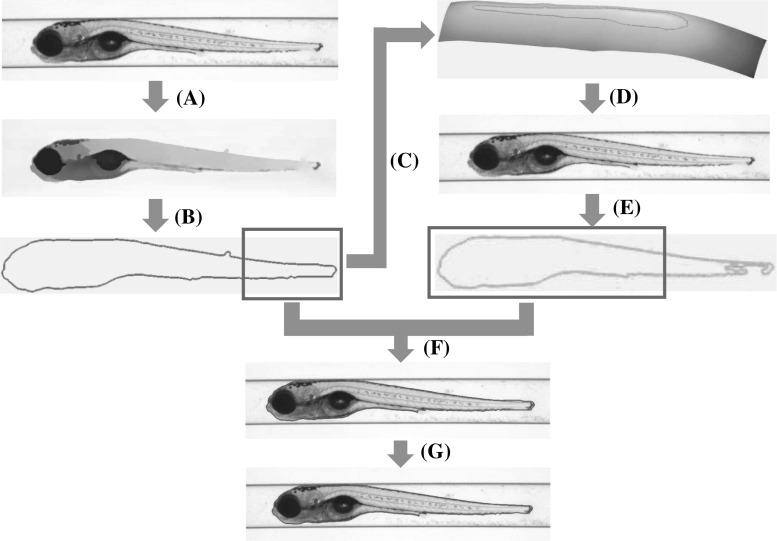
A pipeline schematic of the hybrid method. **a** MS algorithm is applied to improve the visibility of the transparent regions and weak edges. **b** An enclosed contour is extracted from the segmentation candidate in (**a**). **c** A distance-regularised level set function (LSF) is initialised from the zebrafish contour in (**b**). **d** The ILS method is activated and applied on the original image. **e** Another segmentation candidate is generated. **f** An initial hybrid segmentation of the zebrafish is obtained by stitching the remarkable segments from the two candidates according to pre-defined protocols. **g** A refinement is followed to fine-tune the segmentation which can accurately represent the shape of the zebrafish

For this particular research project, we aim at an *efficient* and *robust* solution for accurate zebrafish segmentation from bright-field microscope images. We, therefore, have developed the hybrid (HY) method to combine the advantages of various models. The objective of the HY method is to largely preserve the prominent contour of the object and discriminate the transparent regions and weak edges. In Fig. 2f, we show the segmentation result. A schema of the HY method is depicted in Fig. 3, and below we elaborate the method.

In Fig. 3a, we apply the MS algorithm to the original image to improve the colour representation from the transparent object with respect to the background and obtain a segmentation candidate. This initial segmentation retains and approximates the overall shape of the zebrafish. In Fig. 3b, we extract an enclosed contour for the object from the results obtained in Fig. 3a. In Fig. 3c, a distance-regularised level set function is initialised from the result obtained in Fig. 3b. In Fig. 3d, with the initialised level set function, the ILS method is applied on the original image to obtain another segmentation candidate. It is important that this manner of initialisation significantly accelerates the curve convergence of the level set method and improves the segmentation accuracy. Because the initialisation already approaches the edge potentials, local minimum problem is solved to a certain extent. In Fig. 3f, according to pre-defined protocols, we heuristically fuse the two segmentation candidates. In Fig. 3g, a cascaded refinement module aims to fine-tune the segmentation result, which drives the contour to describe the shape of the zebrafish in a compact and accurate form.

A similar initialisation idea to step (C) is proposed in [14]. However, the employment of the MS algorithm in this work is not only to accelerate and stabilise the curve evolution, but also to obtain an overall view of the shape of the zebrafish which is beneficial for the following hybrid result. In other words, compared to the problem presented in [14], our zebrafish segmentation problem presents a more challenging task; the segmentation methods with just the improved initialisation are insufficient to achieve the best performance.

The remainder of this paper is structured as follows. In Sect. 2, we review the related work and derive the level set method. In Sect. 3, we elaborate the HY method. In Sect. 4, we first present two datasets of zebrafish objects from bright-field microscope imaging. The experimental set-up is subsequently depicted, and the experimental results to evaluate the performance of the proposed method are presented. In Sect. 5, we summarise the research and indicate future developments.

## Related work and inference of level set method

In medical imaging, the functional-based segmentation methods have been successfully developed and shown good performance. These methods seem to be suitable for bright-field microscopy imaging where complex scenes and noise are common. These methods aim at optimising an energy functional to estimate the optimal enclosed contour attaching the object boundary.

An early version of this technique is proposed as the classic active contours (snakes) model [15], from which the more advanced algorithms have been derived. The snakes model detects the object boundary by parameterising it as an enclosed curve $\mathcal {C}(p)\in {\mathbf {R}}^2, ~p\in [0,1]$. The curve will topographically evolve to minimise an energy functional formulated as $E({\mathcal {C}})$ which incorporates an internal force considering the total length and the smoothness of the curve, and an external force derived from the image to encourage the curve to approach the object boundary. However, the snakes model cannot deal with changes in topology; in other words, it cannot detect all the boundaries in an image with multiple objects. Moreover, this method is rather sensitive to blurred edges.

The level set method is developed to handle the problems of topological merging and breaking [16]. The idea is to formulate the object boundary as the zero level set contour implicitly embedded in a three-dimensional function which is known as the level set function (LSF) $\phi ({\mathbf {x}},t):~\varOmega \rightarrow R$, where the *t* is an artificial time variable presenting the time evolution procedure and the $\varOmega$ is the image domain. The $\phi$ is usually assigned with positive and negative values in and out of the zero level set contour. The energy functional is transformed to ${\widehat{E}}(\phi )$ from $E({\mathcal {C}})$.

Subsequently, a region-based level set (CV) model is proposed [10]. With the introduction of the Heaviside function

1 $$\begin{aligned} H(x)=\left\{ \begin{array}{ll} 1, &{}\quad \text {if}\quad ~x \ge 0\\ 0, &{}\quad \text {if} \quad ~x < 0, \end{array} \right. \end{aligned}$$

the energy functional is defined as

2 $$\begin{aligned} \begin{aligned} {\widehat{E}}(\phi )~&= ~ \mu \underbrace{\int _{\varOmega } |\nabla H(\phi )| \mathrm{d}{\mathbf {x}}}_{\text {Length term}}\\&\quad + \,\upsilon \underbrace{\int _{\varOmega } \left( |I-u_{\mathrm{in}}|^2H(\phi )\mathrm{d}{\mathbf {x}} ~+~ |I-u_{\mathrm{out}}|^2(1-H(\phi )) \right) \mathrm{d}{\mathbf {x}}}_{\text {External force}}, \end{aligned} \end{aligned}$$

where $u_{\mathrm{in}}$ and $u_{\mathrm{out}}$ represent the mean intensity of the image inside and outside of the curve, and $\mu$ and $\upsilon$ are constants which can be tuned to balance different forces. The CV model can deal with the edge-blurred images without employing edge terms. Based on the Euler–Lagrange equation, the gradient descent can solve the curve evolution problem. The gradient flow is computed as follows:

3 $$\begin{aligned} \frac{\partial \phi }{\partial t} = - \frac{\partial {\widehat{E}}}{\partial \phi }. \end{aligned}$$

However, as shown in Fig. 1b, the CV model fails to segment the zebrafish because of severe intensity inhomogeneity in the images. A local region-based level set (LRLS) method is proposed to model the intensity variation as a bias term for each of the local region generated from intensity clustering [11].

Differently, the geodesic active contours (GAC) model [9, 17] which originates from the snakes model has its advantage of edge preserving, of which the energy functional is proposed as

4 $$\begin{aligned} \begin{aligned} {\widehat{E}}(\phi )&=\underbrace{\mu \int _\varOmega g(|\nabla I|)|\nabla H(\phi )|\mathrm{d}{\mathbf {x}}}_{\text {Length term}}~\\&+~\underbrace{\upsilon \int _\varOmega g(|\nabla I|)H(\phi )\mathrm{d}{\mathbf {x}}}_{\text {Area term}}\\&=\mu \int _\varOmega g(|\nabla I|)\delta (\phi ) |\nabla \phi | \mathrm{d}{\mathbf {x}}~\\&+~\upsilon \int _\varOmega g(|\nabla I|)H(\phi )\mathrm{d}{\mathbf {x}}, \end{aligned} \end{aligned}$$

where the *g* is known as the edge indicator which is formulated as

5 $$\begin{aligned} g(|\nabla I|) = \frac{1}{1-c|\nabla I|^2}. \end{aligned}$$

The values of *g* are close to zero at the region of object edges and one at the region of non-edges. This definition encourages the curve to converge at the object boundary when the energy functional is minimised. To derive the level set-based GAC model, the gradient flow can be computed according to Eq. (3) as:

6 $$\begin{aligned} \begin{aligned} \frac{\partial \phi }{\partial t}&= \mu \delta (\phi ) \mathrm{div} \left( g(|\nabla I|) \frac{\nabla \phi }{|\nabla \phi |} \right) ~ + ~ \upsilon g(|\nabla I|)\} \delta (\phi )\\&= \mu \delta (\phi )\left[ g(|\nabla I|) \mathrm{div} \left( \frac{\nabla \phi }{|\nabla \phi |} \right) + ~ \nabla g(|\nabla I|) |\nabla \phi |\right] \\&\quad +\, \upsilon g(|\nabla I|) \delta (\phi ). \end{aligned} \end{aligned}$$

Finally, the curve evolution problem is transformed as a level set surface evolution problem

7 $$\begin{aligned} \phi _{i+1} = \phi _i + \Delta t \frac{\partial \phi }{\partial t}, \end{aligned}$$

where the step size controller of $\Delta t$ is tunable during solution search. This search is a standard gradient descent approach which can quickly locate the minimum of the functional.

From the observations of our bright-field images, the contour of the zebrafish is more discriminative than the colour. So, the edge-based level set method should be suitable for our problem. However, from Fig. 1a, c, d, the boundary defects of zebrafish result in the problem of edge leakage for the aforementioned methods. To solve this problem, the shape prior-based level set methods are proposed [18–20]. This type of methods uses pre-defined shape templates to constrain the curve evolution. The employment of the shape constraint enforces the curve to approach the linear transformed template. However, the methods can only deal with the problems with limited shape deformations. Moreover, the methods including curvature constraint try to minimise the total curvature of the curve in order to control curve smoothness [21, 22]. However, these methods are difficult to implement with numerical solutions.

Besides, the performance of the GAC model also depends on the initialisation of LSF. A bad initialiser may lead the curve to converge at a local minimum, for example, the boundaries of the capillary as present in the images of the zebrafish. Cohen and Chen [23, 24] propose to find the global minimum of the geodesic energy by solving the eikonal equation, but those methods require initial and end points from user input. In zebrafish high-throughput imaging, we prefer an automated manner.

Unsupervised learning-based methods, e.g. k-means clustering [25, 26], superpixels [27, 28] and mean shift algorithm [13, 29], represent also a broad category of image segmentation techniques. Those methods can cope with complicated images by merging similar local regions and offer reasonable pre-segmentations.

Supervised learning-based models [30–32] have drawn a lot of attention. Based on the remarkable development of deep learning architectures [33], the fully convolutional neural networks (FCN) [34] have been proposed and they achieved promising performances in semantic segmentation. Consequently, more architectures are proposed [35–37]. Those methods can be seen as generic for the objects which are included in the annotated datasets. Once they are applied in an unseen scenario, a certain number of manual annotations should be prepared, which is usually laboringly and financially expensive. We also have to take the computation complexity into consideration. The FCN usually requires very expensive computation during training time. At inference time, it is inefficient in the scenario without GPU support.

Based on the discussions so far, we may conclude that each of the image segmentation methods shows good properties to solve a generic problem, but also has its own limitations. Therefore, it is reasonable to develop a method to take advantage of the good properties of the methods. Here we aim at an efficient and robust solution for our zebrafish segmentation problem from bright-field microscope images. Considering the intrinsic characteristics of bright-field images of zebrafish, we propose the HY method. This method applies an unsupervised learning method, i.e. mean shift algorithm, to obtain an overview shape description of the object. The edge-based level set method takes the pre-segmentation as initialisation and detects the explicit boundary. Finally, the two segmentation candidates are incorporated to obtain a better shape representation of the zebrafish. In fact, our method can be easily adapted and extended for other similar applications in microscope image segmentation, which does not require many manual interventions.

## The hybrid method for segmentation of zebrafish objects from the bright-field microscope images

In this section we develop the HY method by fusing the advantages of the MS algorithm and the edge-based level set methods, i.e. the ILS method, to obtain accurate segmentation for bright-field microscope imaging of zebrafish. The term *hybrid* represents a dual semantics. We first refer to hybrid as the improved manner of initialisation for the level set method with the MS algorithm. Compared with the functional-based models, the MS algorithm shows the advantage of fast convergence and robust discrimination of transparency and weak edges. In this manner a segmentation candidate representing an overview of the zebrafish shape can be obtained and used to initialise the LSF for the ILS method. The ILS method can obtain another segmentation candidate to retain the explicit contour of the zebrafish. Then we refer to hybrid as the hybrid operation of the two segmentation candidates.

### Mean shift algorithm and the segmentation candidate

We present a short recap of the MS algorithm in the application of clustering. In principle, the MS algorithm can cluster the similar data points through the estimation of the maximal density distribution of each data point. It is a kernel-based density estimator which is derived from a method known as Parzen window. Given *n* data points $\mathbf{x }_i,~i=1,\ldots ,n$, the density distribution of a data point of $\mathbf{x }$ can be approximated by a kernel density estimator as

8 $$\begin{aligned} \hat{f}(\mathbf{x }) = \frac{1}{nh^d} \sum _{i=1}^n K \left( \frac{\mathbf{x }-\mathbf{x }_i}{h}\right) , \end{aligned}$$

where *h* is the size of the bandwidth; *d* is the feature dimension; and $K(\cdot )$ usually takes the form of multivariate Gaussian kernel which can be written as $K(\mathbf{x }) = (2\pi )^{-d/2}\exp (-||\mathbf{x }||^2/2)$. From the definition of Eq. (8), one can find that a data point similar to $\mathbf{x }$ will contribute to its density estimation. We take the profile notation $k(x) = \exp (-x/2)$ instead of the kernel representation of *K* and yields the profile representation of Eq. (8).

9 $$\begin{aligned} \hat{f}_{h,K}(\mathbf{x }) = \frac{c_{k,d}}{nh^d} \sum _{i=1}^n k \left( \left| \left| \frac{\mathbf{x }-\mathbf{x }_i}{h}\right| \right| ^2\right) . \end{aligned}$$

If a function is defined as $g(x) = -k'(x)$, the negative gradient of the profile function *k*, the gradient of (9) can be computed and transformed into the form as follows:

10 $$\begin{aligned} \hat{\nabla } f_{h,K}(\mathbf{x })= & {} \frac{2c_{k,d}}{nh_{d+2}}\left[ \sum _{i = 1}^n g\left( \left| \left| \frac{\mathbf{x }-\mathbf{x }_i}{h} \right| \right| ^2 \right) \right] \nonumber \\&\times \,\left[ \frac{\sum _{i=1:n}\mathbf{x }_i g\left( \left| \left| \frac{\mathbf{x }-\mathbf{x }_i}{h} \right| \right| ^2 \right) }{\sum _{i=1}^n g\left( \left| \left| \frac{\mathbf{x }-\mathbf{x }_i}{h} \right| \right| ^2 \right) } - \mathbf{x } \right] . \end{aligned}$$

The second term in Eq. (10) inspired us to the definition of the *mean shift*

11 $$\begin{aligned} \mathbf{m }_h(\mathbf{x }) = \frac{\sum _{i=1}^n\mathbf{x }_i g\left( \left| \left| \frac{\mathbf{x }-\mathbf{x }_i}{h} \right| \right| ^2 \right) }{\sum _{i=1}^n g\left( \left| \left| \frac{\mathbf{x }-\mathbf{x }_i}{h} \right| \right| ^2 \right) } - \mathbf{x }, \end{aligned}$$

which indicates that the density maximiser of the data point $\mathbf{x }$ directs from the current data point to the kernel-weighted mean of all the training data within a bandwidth of *h*. The location of the maximal density distribution of data point $\mathbf{x }$ can be approximated by updating Eq. (11) until convergence.

We apply the MS algorithm in image texture augmentation which we refer to as the image filtering and smoothing. In our problem of segmentation in images of zebrafish, the texture augmentation is to improve the discrimination from the transparent object with respect to the background and enhance the weak boundary. Considering both the colour and spatial features in images, two bandwidths should be defined separately for those two metrics. The kernel of *K* should combine those two feature spaces and is represented as follows:

12 $$\begin{aligned} K_{h_r,h_s}(\mathbf{x }) = \frac{C}{h_r^3,h_s^2}k\left( \left| \left| \frac{\mathbf{x }^r}{h_r}\right| \right| ^2 \right) k\left( \left| \left| \frac{\mathbf{x }^s}{h_s}\right| \right| ^2 \right) , \end{aligned}$$

where *k* keeps the form of profile as previous definition; $(\mathbf{x }^r,\mathbf{x }^s)$ denote colour and spatial features, respectively; and the pair $(h_r,h_s)$ represents the bandwidth in the two feature spaces. We use the three-channel RGB image and represent the spatial feature as two-dimensional coordinates of the pixel location. According to Eq. (12), the pixels within a range domain contribute more, i.e. represented as higher weights, for the density estimation of the centre pixel when the neighbouring pixels and the centre pixel are similar in colour and spatial space.

By determining a proper combination of the bandwidths for $(h_r,h_s)$ and applying the MS algorithm on the images of zebrafish, the weak boundary of the specimen can be, to a certain extent, recovered by the neighbouring pixels. At the same time, the colour inhomogeneous regions are smoothed. For our application, only one object is present in the image, so a segmentation candidate for the zebrafish is directly obtained by thresholding the texture augmented images and represented as ${\mathcal {S}}_M$.

### The hybrid of the improved level set method and the accelerated initialisation

In this work, we apply the ILS method for two reasons: (1) efficient implementation and (2) its tunable properties to a problem. The ILS method improves the GAC model by the employment of a “region-based term”. Its energy functional is defined in Eq. (13).

13 $$\begin{aligned} {\widehat{E}}(\phi )= \int _\varOmega \left[ \mu g(|\nabla I|)|\nabla H_\epsilon (\phi )| ~+~ \upsilon (I-m) H_\epsilon (\phi ) \right] \mathrm{d}{\mathbf {x}}, \end{aligned}$$

where *m* is a user-provided value which is used to pre-process the images. We use a smooth approximation of the Heaviside function, here defined as

14 $$\begin{aligned} H_\epsilon (x)=\left\{ \begin{array}{lll} \frac{1}{2}\left( 1+\frac{x}{\epsilon }+\frac{1}{\pi }\sin \left( \frac{\pi x}{\epsilon }\right) \right) , &{}\quad \text {if}\quad ~|x| \le \epsilon \\ 1, &{}\quad \text {if}\quad ~x > \epsilon \\ 0, &{}\quad \text {if}\quad ~x < -\epsilon , \end{array} \right. \end{aligned}$$

and its derivative

15 $$\begin{aligned} \delta _\epsilon (x) = \left\{ \begin{array}{lll} \frac{1}{2\epsilon }\left[ 1+\cos \left( \frac{\pi x}{\epsilon }\right) \right] , &{}\quad \text {if}~\quad |x| \le \epsilon \\ 0, &{}\quad \text {if}~\quad |x| > -\epsilon . \end{array} \right. \end{aligned}$$

According to Eq. (3), the gradient flow of the ILS method is derived as:

16 $$\begin{aligned} \frac{\partial \phi }{\partial t}= & {} \delta _\epsilon (\phi )\bigg \{\mu \left[ g(|\nabla I|) \mathrm{div} \left( \frac{\nabla \phi }{|\nabla \phi |} \right) + \nabla g(|\nabla I|) \frac{\nabla \phi }{|\nabla \phi |} \right] \nonumber \\&\quad + \,\upsilon (I-m) \bigg \}, \end{aligned}$$

where div denotes the divergence operator.

Basically, the ILS method replaces the “area constraint” in the original GAC model by a region-based term inferred from the image to make the solution more tunable. For the sake of fast implementation, the additive operator splitting (AOS) scheme [17, 38] is used.

In general, an LSF should be defined to initialise the level set methods. Multiple options are available to accomplish this, e.g. random initialisation. Application of a random initialisation for segmentation of zebrafish images has the risk of the enclosed contour of the zero level set converging at a local minimum which is presented as the noise. The segmentation candidate from the MS algorithm offers an overall shape representation of the zebrafish, which is a reasonable initialiser and can be fed to the ILS method. The LSF initialised by the MS algorithm is an approximation of the object, which imposes the curve evolution of the ILS method to be activated from a considerably good location. Based on this idea, we accomplish the first goal of the HY method and specify the curve evolution of Eq. (7) in two phases:

17 $$\begin{aligned} \left\{ \begin{array}{l} \phi _1 = \phi _0^M + \Delta t_1 \frac{\partial \phi }{\partial t},\quad t=0,\\ \\ \phi _{t+1} = \phi _t + \Delta t_1 \frac{\partial \phi }{\partial t},\quad t=1~\text {to}~T_1-1, \end{array} \right. \end{aligned}$$

where the notation $\phi _0^M$ denotes the shape-constrained LSF by the MS algorithm. Compared to the random initialisation fashions, the proposed HY method leads the LSF to approach the global minimum, such that the ILS method is accelerated and more robust with less iterations. We obtain the second segmentation candidate of the zebrafish, represented as ${\mathcal {S}}_L$ through searching for the non-negative level sets in the converging LSF of $\phi$.

### The hybrid of the segmentation candidates

In order to accomplish the second task of the HY method, we define a hybrid operator to obtain the hybrid for the two segmentation candidates. To that end, we first detect the orientation of the zebrafish. In general, the side close to the broadest part of a zebrafish is recognised as the head side. The hybrid operator includes multiple operations of splitting and fusing and is mathematically defined as

18 $$\begin{aligned} {\mathcal {S}} = \alpha \cdot {\mathcal {S}}_L \oplus \beta \cdot {\mathcal {S}}_M, \end{aligned}$$

where ${\mathcal {S}}, ~{\mathcal {S}}_L, \text {and}~{\mathcal {S}}_M$ represent the segmentations by the hybrid operation, the ILS method and the MS algorithm, respectively. We define $\alpha \in [0,1]$ and $\beta \in [0,1]$ as splitting factors which satisfy the criterion $\alpha +\beta \geqslant 1$. Here, we use $\alpha =\beta =0.9$. This ensures the zebrafish shape integrity.

We implement the splitting operator as $\alpha \cdot {\mathcal {S}} = {\mathcal {S}}_\alpha ^H \cup {\mathcal {S}}_\alpha ^T$, where ${\mathcal {S}}^H$ and ${\mathcal {S}}^T$ denote the segments from the *head* and *tail* sides of the zebrafish. In other words, we separately split the whole zebrafish shape into the head and tail parts according the factor $\alpha$ (and $\beta$).

We have observed that the ILS method offers more compact contour for the segment close to the side of head in zebrafish, so we could take the intersection of the corresponding segments from the two segmentation candidates. The MS algorithm offers an approximation for the natural shape of zebrafish for the segment close to the side of tail, so we take the union of the corresponding segments. As a result, we elaborate Eq. 18 as follows to complete the hybrid operation.

19 $$\begin{aligned} \begin{aligned} {\mathcal {S}}&= \alpha \cdot {\mathcal {S}}_L \oplus \beta \cdot {\mathcal {S}}_M \\&= ({\mathcal {S}}_{L,\alpha }^H \cup {\mathcal {S}}_{L,\alpha }^T \oplus ({\mathcal {S}}_{M,\beta }^H) \cup {\mathcal {S}}_{M,\beta }^T) \\&= ({\mathcal {S}}_{L,\alpha }^H \cap {\mathcal {S}}_{M,\beta }^H) \cup ({\mathcal {S}}_{L,\alpha }^T \cup {\mathcal {S}}_{M, \beta }^T). \end{aligned} \end{aligned}$$

From the observation of the initial result of the HY method, segmentation artefacts at the stitching point might occur. Therefore, we propose a refinement in the form of the second-phase curve evolution based on the LSF initialised by the initial hybrid segmentation result. We specify this idea in Eq. (20). Hereby we use *u* to define the LSF to distinguish from Eq. (7).

20 $$\begin{aligned} \left\{ \begin{array}{l} u_1 = u_0^{{HY}} + \Delta t_2 \frac{\partial u}{\partial t}, \quad t=0,\\ \\ u_{t+1} = u_t + \Delta t_2 \frac{\partial u}{\partial t},\quad t=1~\text {to}~T_2-1. \end{array} \right. \end{aligned}$$

Through the aforementioned manner, we can obtain more accurate representation of the zebrafish contour which is embedded as the zero level set in the *u*. The step size $\Delta t_2$ of the gradient flow is set to be much smaller than the previous one of $\Delta t_1$, which prevents the occurrence of edge leakage. In order to clearly illustrate the proposed method, we summarise the whole procedure in Algorithm 1.
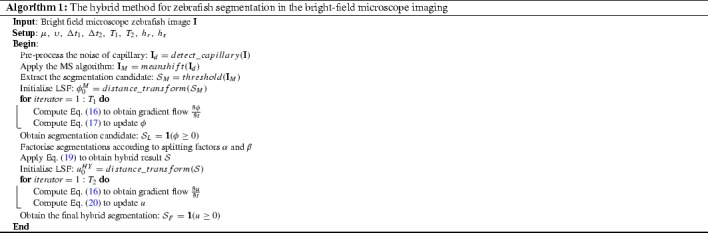


## Experiments

In this section we first present two datasets of bright-field axial-view images of zebrafish from the vertebrate automated screening technology (VAST BioImager) (http://www.unionbio.com/vast/). We apply our HY method as well as several popular segmentation methods on the datasets to compare performances. We evaluate the methods in the form of accuracy and efficiency. The visualisation of segmentation results shows the limitations of the reference methods and the merit of the HY method for segmentation of bright-field microscope images of zebrafish. At the end, we provide an evaluation of the FCN on our datasets and explore the potentials of our method in supporting the FCN.

### Data collection

The VAST BioImager is developed for high-throughput experiments with zebrafish; the device can be mounted on a microscopes; its main feature is the ability of manipulation of zebrafish in the field of view by loading them in capillary. The VAST camera detects the orientation and location of the object. Once the object is present in the field of view of the imager, a set of stepper motors holding the capillary rotate the specimen in a full revolution, so that images of the zebrafish can be acquired in any axial view. In our experiments, 84 axial views (images) are evenly sampled from a full revolution (around $4.3^{\circ }$ per view) for each specimen. This axial-view imaging protocol presents another challenge to the generalisation ability of the segmentation methods.

**Fig. 4:**
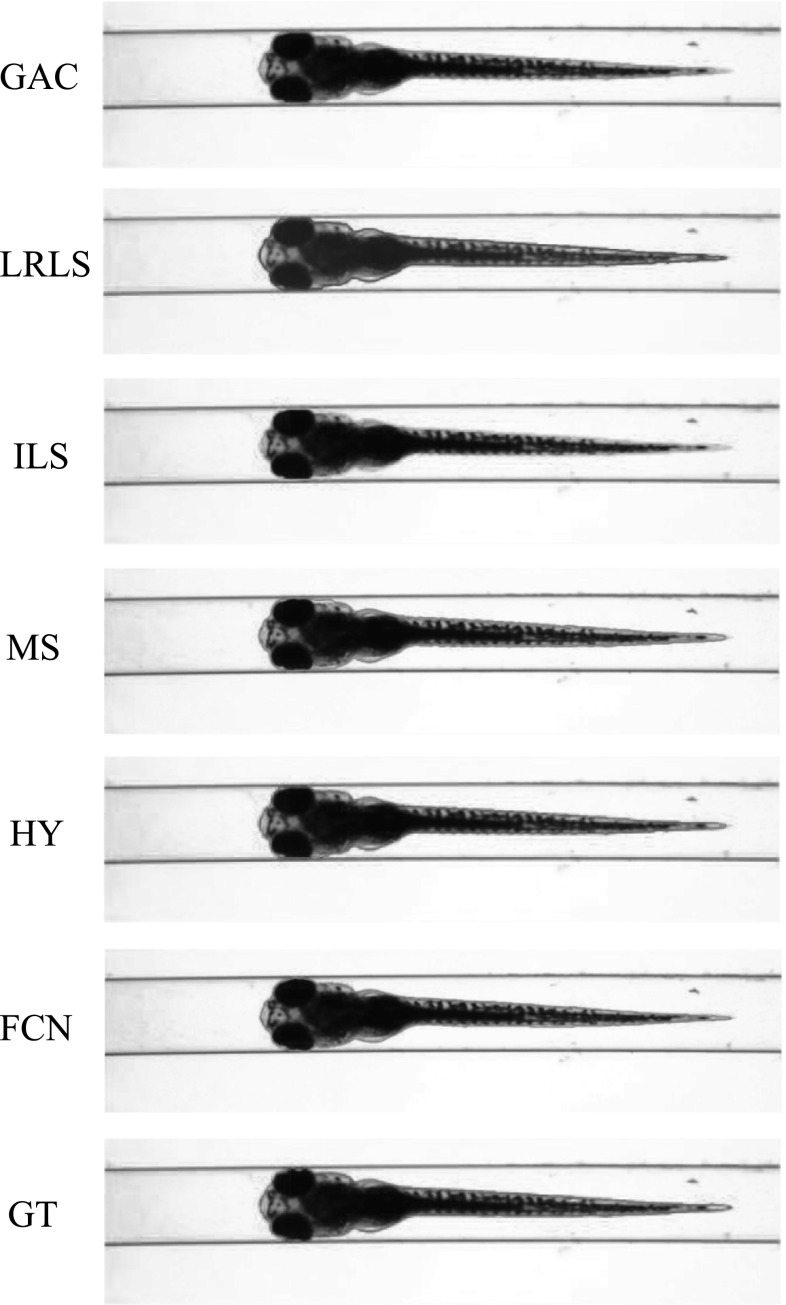
Segmentation results visualisation of different methods on one zebrafish example from *Dataset A*. The object is positioned in ventral. GAC = geodesic active contours model [9]. LRLS = local region-based level set model [11]. ILS = improved level set method [12]. MS = mean shift algorithm [13]. HY = the proposed hybrid method. FCN = fully convolutional neural networks. GT = groundtruth

**Fig. 5:**
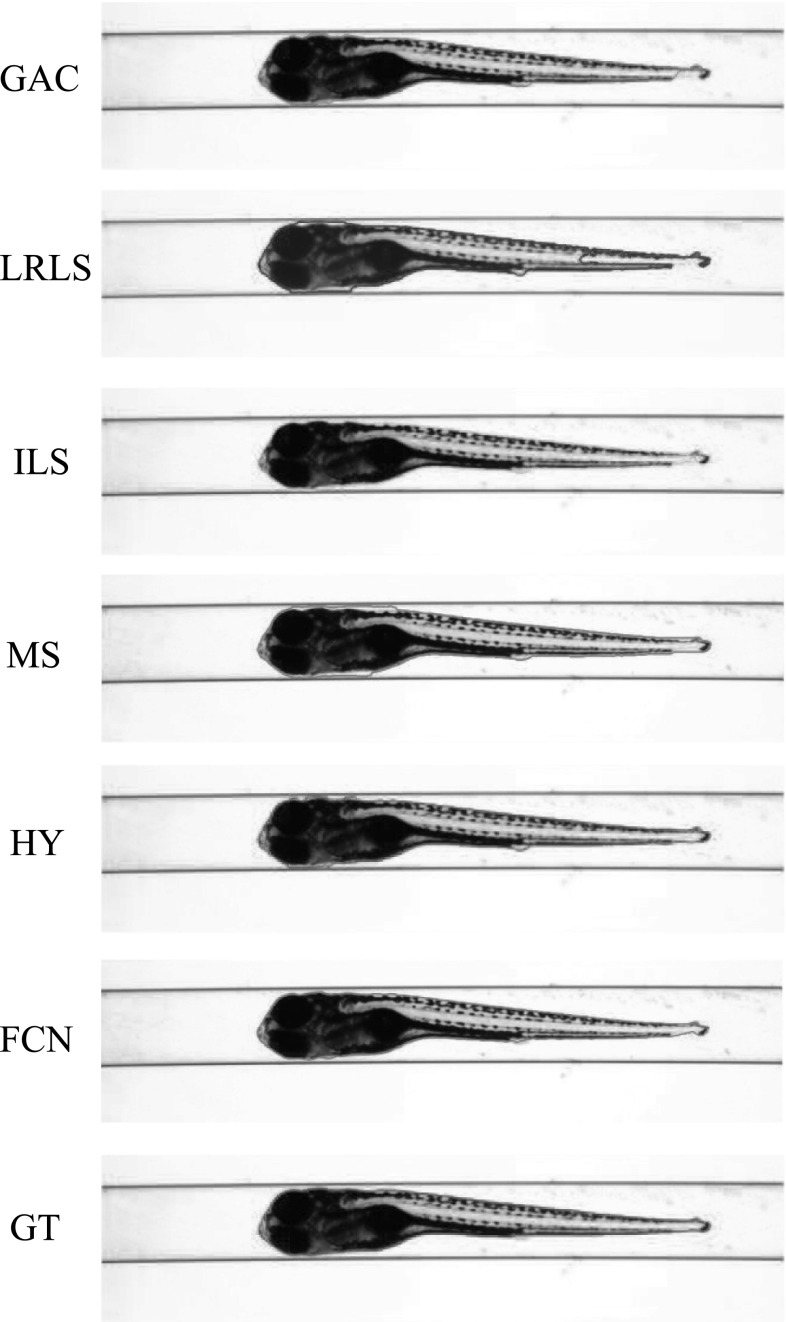
Segmentation results visualisation of different methods on one zebrafish example from *Dataset A*. The object is in titled position

**Fig. 6:**
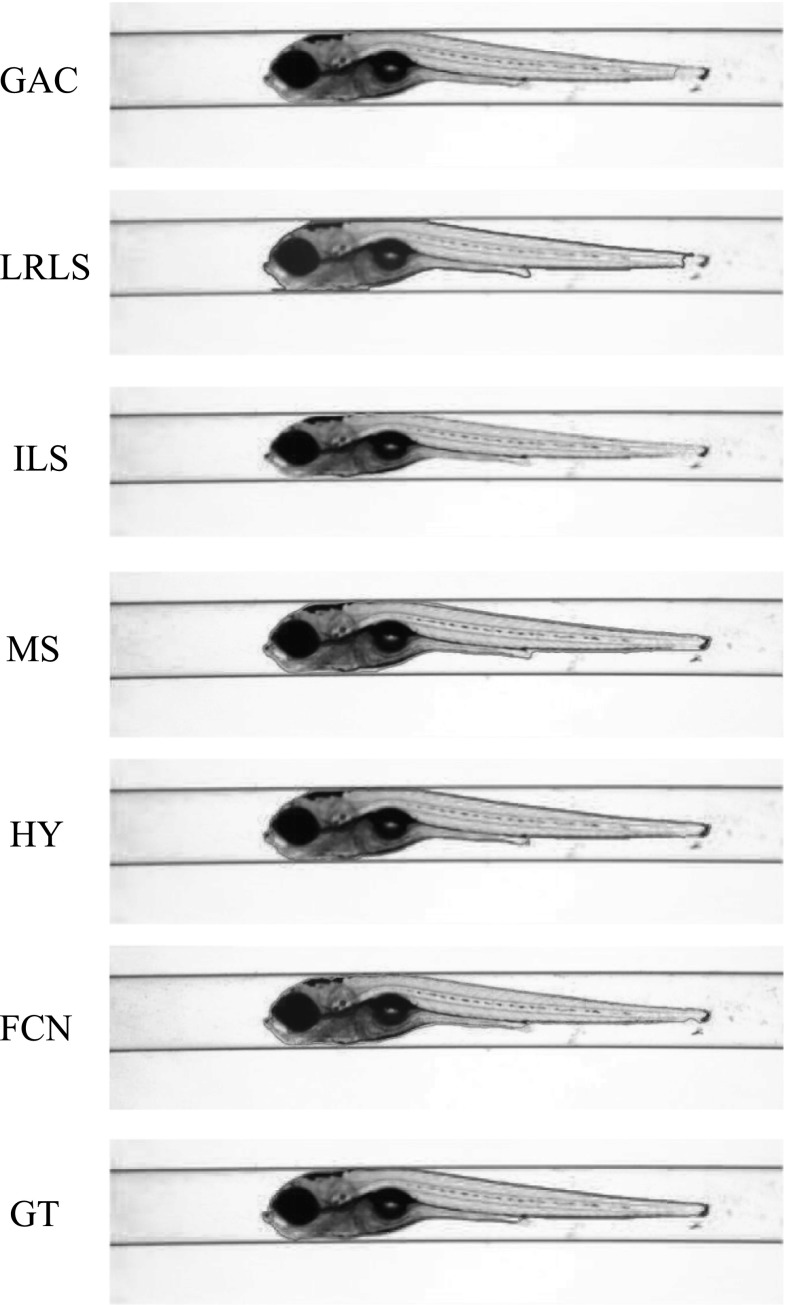
Segmentation results visualisation of different methods on one zebrafish example from *Dataset A*. The object is positioned in lateral

**Fig. 7:**
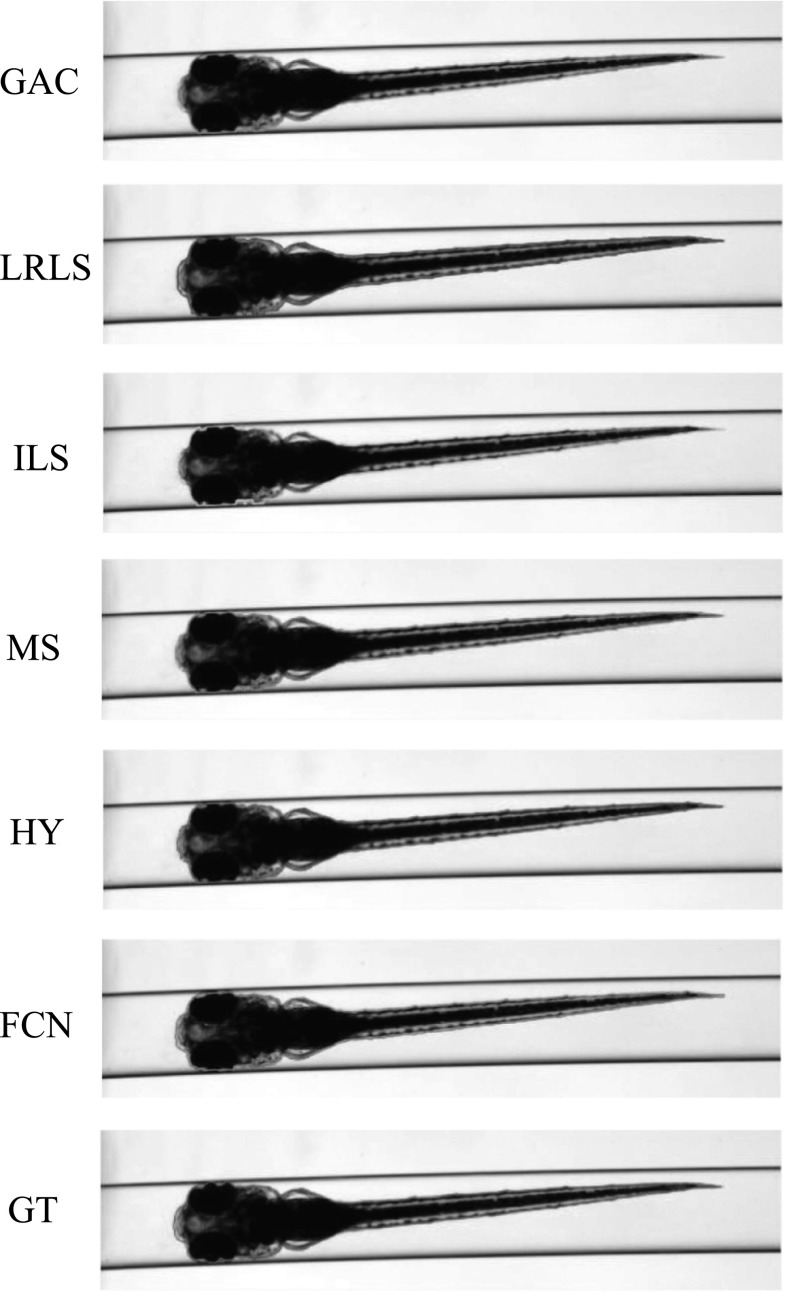
Segmentation results visualisation of different methods on one zebrafish example from *Dataset B*. The object is positioned in ventral

**Fig. 8:**
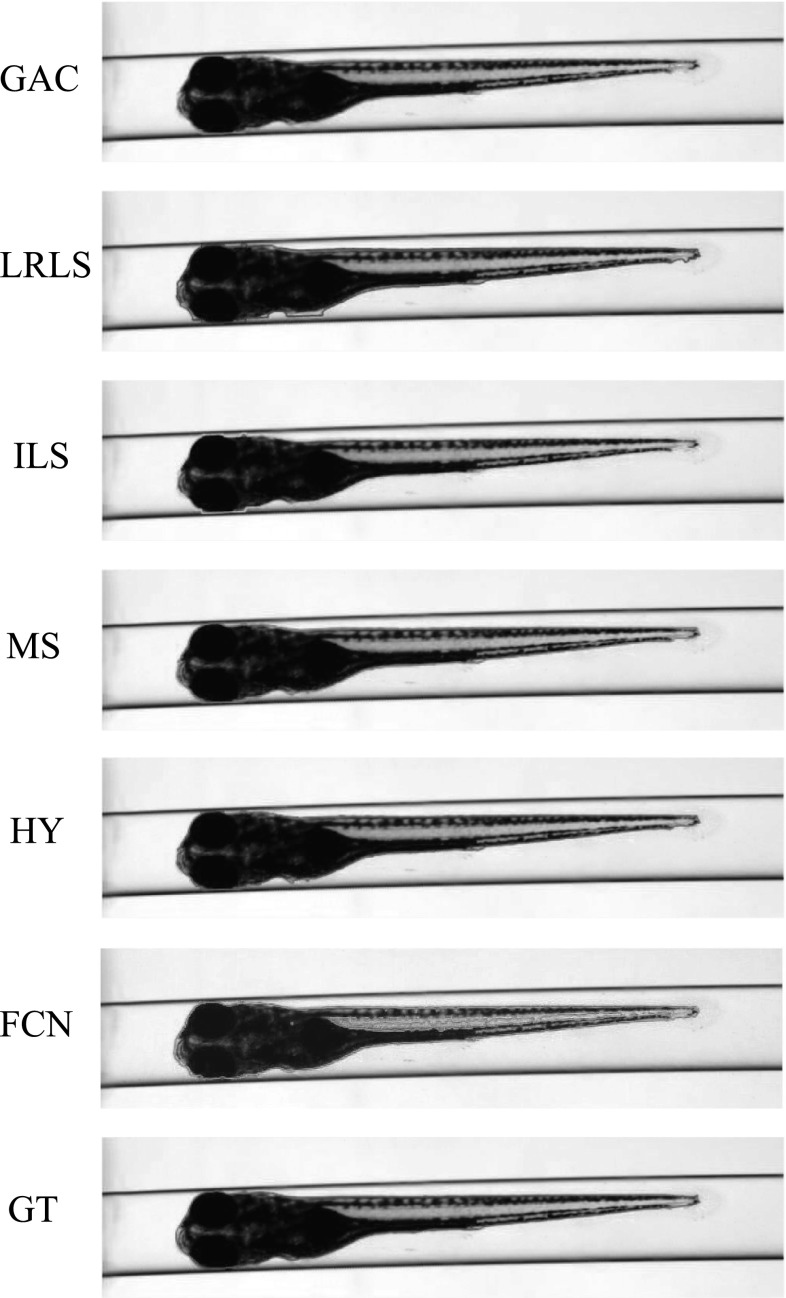
Segmentation results visualisation of different methods on one zebrafish example from *Dataset B*. The object is in tilted position

**Fig. 9:**
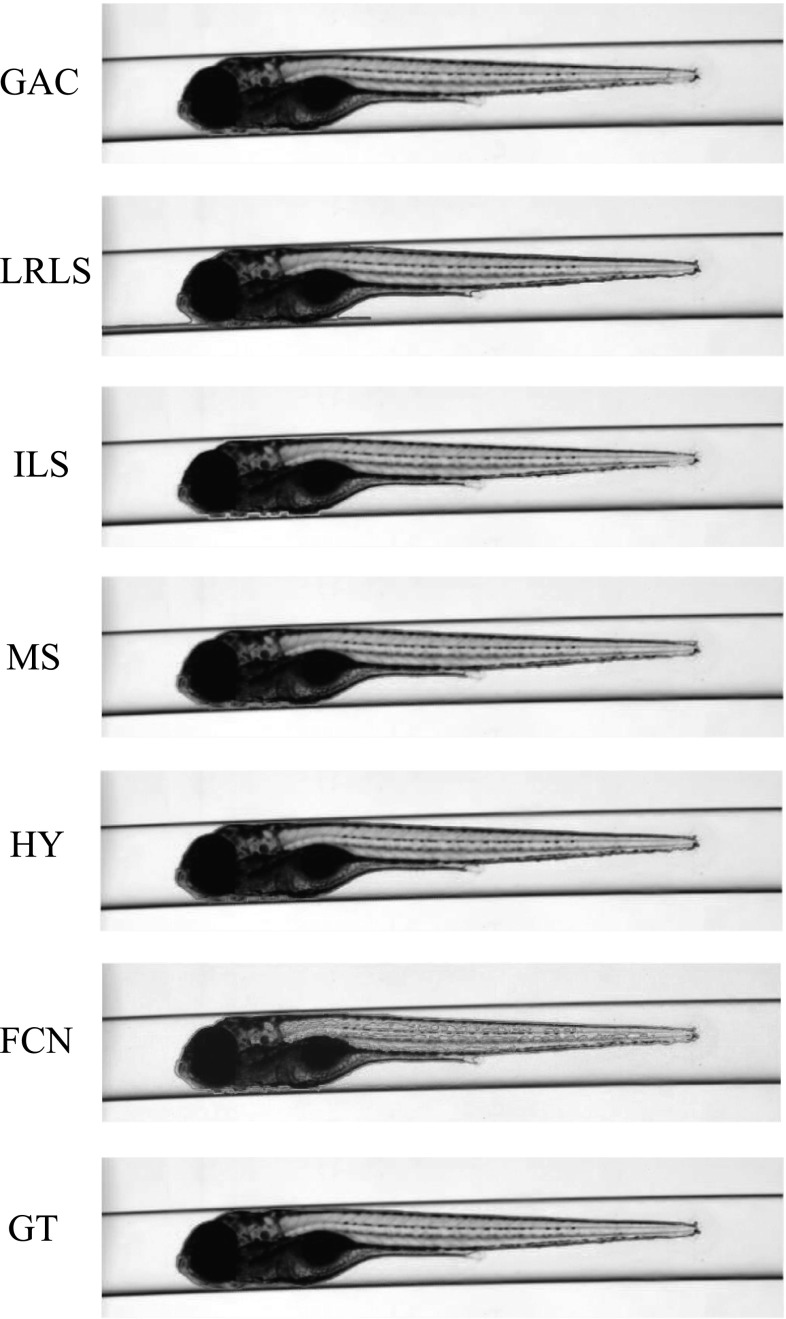
Segmentation results visualisation of different methods on one zebrafish example from *Dataset B*. The object is positioned in lateral

*Dataset A*—The VAST BioImager is equipped with a standard camera, the VAST camera, which is used to detect the object presence in the field of view. With this camera axial-view images for the specimen can also be acquired representing an overview of the object. These images are $1024 \times 1024$ in size with a pixel size of $5.5\times 5.5~\upmu \hbox {m}$. From Figs. 4, 5 and 6, examples of the images acquired by the VAST camera are depicted. The partial transparency and weak edge are clearly visible in most of the images. All images in the *Dataset A* are collected with the VAST camera. *Dataset A* includes a range of developmental stages of the zebrafish, i.e. 3, 4 and 5 days post-fertilisation (dpf). The dataset contains three groups with 60 examples. With 84 views per sample, this results in over 5000 images in total ($84\times 60$).

*Dataset B*—The images produced by the VAST BioImager are of relatively low resolution and are insufficient for detailed observations of the zebrafish. Our set-up consists of a microscope on which the VAST BioImager is mounted to produce high-resolution images. The VAST BioImager manipulates the specimen, and the camera mounted on the microscope acquires the high-resolution images. Therefore, as an extension to *Dataset A* a *Dataset B* is obtained. The same imaging protocol with respect to *Dataset A* is used, i.e. 84 evenly sampled axial views are acquired in a full revolution. The image size of each is $1920 \times 2560$ with a pixel size of $3.4 ~\upmu \hbox {m}\times 3.4~\upmu \hbox {m}$. From Figs. 7, 8 and 9, some examples of these images are depicted. For better visualisation, both of the vertical sides of the images are cropped to the centre of the object and the image size is cropped to $600 \times 2560$.

Here, we state that the segmentation of zebrafish in bright-field microscope images is relevant to the visible parts of the objects. In the zebrafish image examples shown in Figs. 4, 5, 6, 7, 8 and 9, we can see that the caudalmost extension of yolk of the zebrafish positioned at its lateral view is almost entirely invisible. The caudalmost tip of the tail is also invisible at its lateral view, but is visible at its ventral view. However, we should realise that all the methods which will be evaluated cannot recognise those parts without any shape constraints. Moreover, the visible shape of the zebrafish is already sufficient in our applications mentioned in Sect. 1. So, in this work, we only include the visible shapes presenting in the zebrafish images. However, regarding the caudalmost tip of the tail, we can still apply our previous work [39, 40] to handle it. For example, we first create a 3D model for the zebrafish using the segmentations obtained in this work and project it back to the 2D shapes to improve the segmentations. Reader can refer to our previous work for more details.

### Evaluation of different methods

In the experiment, the efficiency and performance are evaluated for different segmentation methods. The abbreviations of *CV*, *GAC*, *LRLS* and *ILS* consistently represent the Chan–Vese model, geodesic active contours model, local region-based level set model [11] and the improved level set method [12], respectively; *MS* denotes the mean shift algorithm. The representation of $*+$*MS* indicates the $*$ model with an initialiser from the MS algorithm, and *HY* is the proposed HY method.

In order to have a groundtruth set, we manually segmented 336 images of 4 specimens (84 views per specimen) from *Dataset A*. In addition, a subset from *Dataset B* including 33 images selected from 3 objects is also manually segmented to obtain groundtruth annotations.

We measure the accuracy represented as *F*-score and the efficiency as run-time for all the methods on the subsets. The *F*-score is defined as $F = (2\cdot recall \cdot precision)/(recall+precision)$. The closer to one the *F*-score is, the better the performance of a method is. The mean and standard deviation for the two measurements are computed.

In the experiment, we partially used the fast implementation from [12]. To justify different methods, we give the same set-ups. For the models initialised by the MS algorithm, we take the configuration of the kernel bandwidths $(h_r,h_s)$ as (20, 20). Besides, all the methods are configured with the same number of iterations.

#### Performance evaluation on subsets of *Dataset A*

In Table 1, we show the performance of different methods, evaluated on the subset of *Dataset A* with groundtruth. One can see that the CV model obtains the lowest *F*-score. This can also be seen in the segmentation result visualisation depicted in Fig. 1b. Due to intensity inhomogeneity of the zebrafish in the image, it is difficult for the CV model to estimate the general mean of the texture inside and outside the object. Consequently, the CV model almost completely fails to detect the zebrafish.

For the other methods, comparable performances are seen though; differences are still existing. It is obvious that the MS algorithm is the most efficient segmentation method. This provides evidence for the fact that a segmentation method equipped with an MS initialiser is always more efficient than the same model with the random initialisation. We may conclude that the hybrid of the MS initialisation with the functional-based segmentation model is helpful to improve the efficiency of zebrafish segmentation. The reason is that the MS initialiser can produce a good estimation of the overall shape of the zebrafish. This shape approaches the global minimum.

The LRLS model also achieves a good performance. However, we should make more effort for the configurations and post-processing to obtain a natural shape for the zebrafish in the LRLS model. We do not have the fast implementation for the LRLS model, so that we cannot reasonably give a justification of its efficiency. Nevertheless, we can appreciate the hybrid of the MS algorithm and the LRLS model for a fast curve evolution.

Both the ILS method and the GAC model can obtain better segmentation results than the aforementioned methods. We find that the ILS method works faster than the GAC model. So, we choose to use the ILS method in our HY method. Considering the accuracy, the proposed HY method has the best performance. This is reasonable as the HY method combines the advantages of the MS algorithm and the ILS method. The segmentation result preserves an overall shape and retains the original explicit contour of the zebrafish.

#### Performance evaluation on *Dataset B*

In Table 2 we show the performances of the different methods as evaluated on the subset of *Dataset B*. We can directly see that the efficiency of all methods is lower as a result of the larger image size. In addition, similar to the experiment on *Dataset A* it can be seen that the methods equipped with the MS initialiser generally work faster than the methods with random initialisation. Although the LRLS model obtains slightly better results than the ILS, the latter usually works faster. We do not have equivalent implementation of the LRLS model, so for the run-time, no justification can be given. Due to the employment of the hybrid operation and post-processing, the proposed HY method works a little bit slower than the ILS method with an MS initialiser, but the segmentation accuracy is clearly improved.

**Table 1:** Comparison of different methods on *Dataset A*

Model	Run-time (s)	*F*-score
CV model	$1.74 \pm 0.31$	$0.758 \pm 0.123$
CV model+MS	$1.32 \pm 0.16$	$0.758 \pm 0.123$
LRLS	$22.83 \pm 3.70$	$0.956 \pm 0.026$
LRLS+MS	$19.56 \pm 0.15$	$0.968 \pm 0.014$
GAC model	$3.34 \pm 0.38$	$0.976 \pm 0.006$
GAC model+MS	$1.72 \pm 0.13$	$0.976 \pm 0.007$
ILS	$2.65 \pm 0.42$	$0.976 \pm 0.007$
ILS+MS	$1.26 \pm 0.32$	$0.978 \pm 0.006$
MS	$0.63 \pm 0.07$	$0.964 \pm 0.006$
HY	$1.37 \pm 0.22$	$\mathbf {0.983} \pm \mathbf {0.004}$

The best performance among different methods is shown in bold

**Table 2:** Comparison of different methods on *Dataset B*

Model	Run-time (s)	*F*-score
CV model	$8.87 \pm 1.78$	$0.838 \pm 0.120$
CV model+MS	$6.96 \pm 1.63$	$0.838 \pm 0.120$
LRLS	$152.27 \pm 1.06$	$0.968 \pm 0.016$
LRLS+MS	$126.60 \pm 1.76$	$0.977 \pm 0.011$
GAC model	$21.92 \pm 0.19$	$0.918 \pm 0.068$
GAC model+MS	$8.95 \pm 0.40$	$0.957 \pm 0.034$
ILS	$14.53 \pm 6.39$	$0.970 \pm 0.015$
ILS+MS	$7.23 \pm 1.73$	$0.973 \pm 0.022$
MS	$2.32 \pm 0.31$	$0.965 \pm 0.023$
HY	$8.30 \pm 0.98$	$\mathbf {0.986} \pm \mathbf {0}.\mathbf {004}$

The best performance among different methods is shown in bold

### Inspection of results by visualisation

In this experiment, we have visualised some representative segmentation results of *Dataset A* and *Dataset B* in this experiment.

**Fig. 10:**
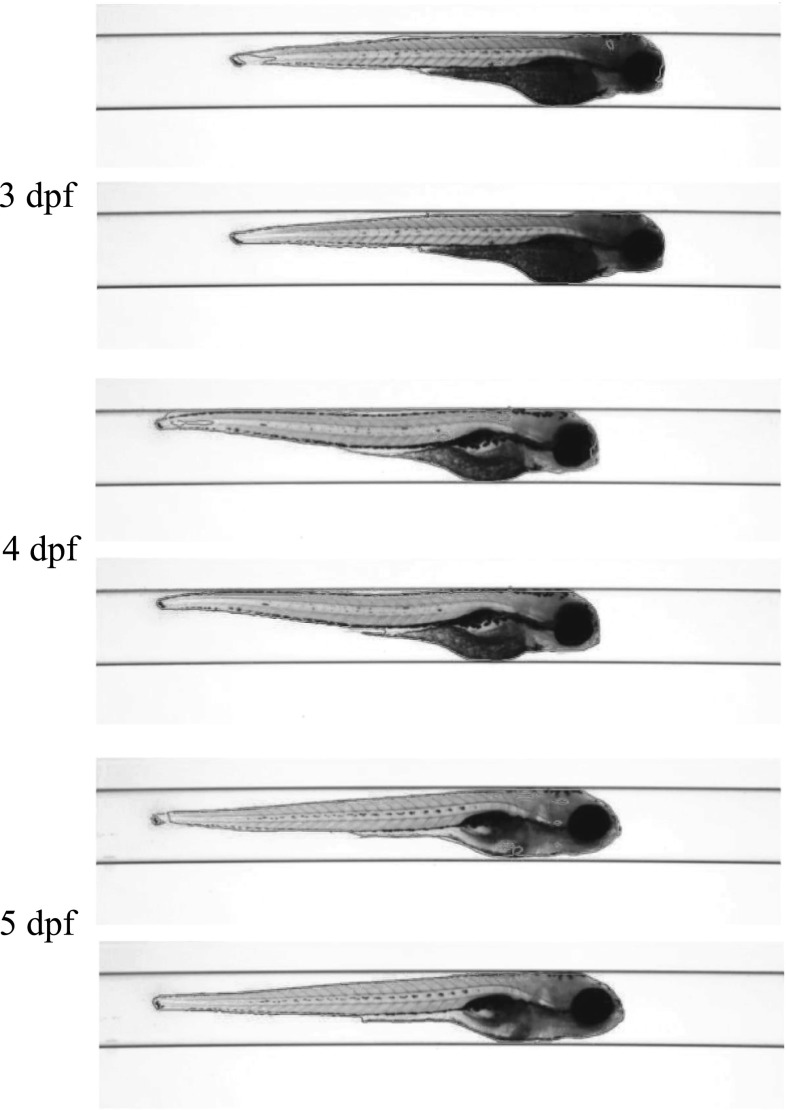
Segmentation results visualisation on *Dataset A*. We randomly select one subject from each of the three larval stages and show the lateral view. In each bounding box, the upper figure shows the result obtained from the FCN trained from our annotated subset of *Dataset A*. And the bottom figure shows the result obtained from our HY method

For *Dataset A*, we randomly selected one zebrafish specimen from the annotated subset of *Dataset A*. We show three typical axial views (lateral, $45^{\circ }$ tilted and ventral) in Figs. 4, 5 and 6. We can observe that for the images with the zebrafish positioned in the view of ventral (dorsal), all the methods result in an accurate segmentation; this is due to the fact that the image portrays an explicit boundary of zebrafish. In the images with a lateral view of the zebrafish, the GAC model, LRLS model and ILS method fail to detect the weak edges. This phenomenon of edge leakage commonly occurs. Although the MS algorithm can retain a natural shape for the zebrafish, it loses the edge sensitivity. The proposed HY method obtains more accurate segmentations. In order to illustrate the generalisation of method, we select another three subjects from each developmental group in *Dataset A* and visualise the segmentation results in Fig. 10. (The subjects are shown in lateral view.)

From Figs. 7, 8 and 9, a representative subject from *Dataset B* positioned in three typical axial views is depicted. Compared to *Dataset A*, these images have a better contrast and the outline (contour) of the zebrafish specimen is more explicit. Consequently, the classical edge-based segmentation methods such as the GAC model have less difficulty segmenting the zebrafish from these images. The risk of edge leakage, however, still exists. In Figs. 8 and 9, we can see the contours resulted from the GAC model, LRLS model and ILS method converging at the wrong regions. The MS algorithm results in a segmentation retaining the whole boundary of the object, but the shape as a whole is less compact. From our experiment, we may conclude that the proposed HY method is able to deal with the segmentation problem for zebrafish specimens in bright-field microscopy. It results in more accurate results and shows a good performance. Due to the illumination conditions in the microscope, the acquired images are sometimes less explicit; this is depicted in the third column of Fig. 1a. A straightforward pre-processing solution such as colour equalisation can improve the image contrast of the object with respect to the background. More segmentation results in this experiment represented as animations can be found here: http://bio-imaging.liacs.nl/galleries/VAST-Hybrid/.

### Exploration on convolutional neural networks

In this experiment, we evaluate the performance of the FCN on our datasets. (For details of the FCN refer to the original work [34].) We should note that this evaluation cannot directly show the performance comparison with our method, because the FCN is a supervised learning-based method, while our method is categorised as an unsupervised learning-based method.

We design three strategies to enable the experiment. (A) We use three of the annotated subjects of *Dataset A* as training set and the left one for testing. (B) We use the four annotated subjects of *Dataset A* to train the FCN and then test on the whole *Dataset A* (except the four annotated subjects). In this case, we do not have groundtruth for the performance evaluation, so we use the segmentation results obtained by our method as “groundtruth” approximation. The rationale behind this is the validated performance of our method. (C) We use the same model trained in (B) to test on *Dataset B*. We aim to investigate the generalisation of the FCN. The objects in *Dataset B* are not entirely the same as in *Dataset A*, but they still share considerable similarity, e.g. shape and textures of the object, imaging conditions. For the three strategies, we use the default settings at training time, e.g. iterations, learning rate, pre-processing. At testing time, we use the CPU mode as used in our method.

**Table 3:** Performance evaluation of the FCN

Strategy	Run-time (seconds)	*F*-score
Strategy (A)	$16.7 \pm 0.3$	$0.984 \pm 0.003$
Strategy (B)	$16.2 \pm 0.4$	$0.969 \pm 0.009$
Strategy (C)	$71.6 \pm 0.5$	$0.948 \pm 0.016$

Italics represents that we do not have groundtruth to measure the F-score in strategy B. Instead we use the results from our method as a reference. So this measurement is not totally objective and precise

In Table 3, we report the results from which we may get the following clues. (1) We can find that our method, as an unsupervised method, can obtain promising segmentation results as a fully supervised method, e.g. the FCN. We visualise the result obtained by the FCN in strategy (A) in Figs. 4, 5 and 6. (2) Our method may outperform the FCN which are provided with a limited amount of training examples on the whole *Dataset A*. We select three examples and visualise the results in Fig. 10. In order to improve the performance of the FCN on the whole *Dataset A*, we design another strategy. This strategy uses the segmentation results obtained by our method as groundtruth approximation and selects $50\%$ of the subjects to augment the training and the left half for testing. We can obtain the F1-score of $0.986 \pm 0.003$. (3) For an unseen scenario which still retains a large quantity of similarities of the training examples, the FCN can, to a limited extent, recognise the object. From Figs. 7, 8 and 9, we can see the segmentation results with lots of noise. (4) Regarding the efficiency evaluated with CPU mode, our method is more than 10 times faster than the FCN for inference. We admit that the FCN can obtain much faster performance with GPU support, but in this work, we do not have an equivalent evaluation of our method. (5) Therefore, we may draw an important conclusion that our method can be used to obtain groundtruth approximation for training the FCN.

## Conclusions and future work

We have presented a hybrid method to accomplish the task of efficient and accurate segmentation of zebrafish from the bright-field microscope images. We propose to employ the mean shift algorithm to augment the colour representation for the partial transparent regions and transform the ambiguous edges more separable, such that we can obtain a segmentation candidate which preserves an overview of the zebrafish shape. A distance-regularised level set function is initialised from this segmentation candidate and fed to an improved level set method in order to obtain a more compact shape representation preserving the explicit object contours. This hybrid operation accelerates the curve convergence at the regions of interest. We intuitively fuse those two segmentation candidates and employ a refinement in order to obtain the accurate hybrid segmentation. The results of our segmentation method facilitate the visualisation and evaluation of gene expressions in zebrafish in both 2D and 3D. This is directly relevant to the success of experiments in which imaging is crucial. Such experiments are typical for applications in life sciences, e.g. cancer and pharmacokinetics. Furthermore, the proposed method is very suitable for high-throughput applications with zebrafish.

The proposed method can be generalised by taking images into consideration that contain multiple objects positioned in various orientations. For orientation detection and initialisation over multiple instances modules need be developed that constitute the generalisation. For the work presented in this paper, the single instance is the approach for high-throughput applications. Moreover, bright-field microscopy is a standard component for this type of applications. Nevertheless, the proposed HY method can be evaluated for other imaging modalities, with other lenses and illumination architectures. In this manner the HY method is probed and challenged for other and different image qualities. As an example, we consider optical projection tomography (OPT) imaging [41]; bright-field images are included in this imaging technique and the processing of these images might benefit from the application of the proposed HY method. We find that our method can be used to produce the segmentations as groundtruth shape approximation. In biomedical image processing, this will lead to less manual dedications in annotation. The results can be used for training deep convolutional neural networks. This will be further verified on an extension of our *Dataset B*.
